# Intraperitoneal Administration of Oxygen/Ozone to Rats Reduces the Pancreatic Damage Induced by Streptozotocin

**DOI:** 10.3390/biology7010010

**Published:** 2018-01-11

**Authors:** Dario Siniscalco, Maria Consiglia Trotta, Anna Lisa Brigida, Rosa Maisto, Margherita Luongo, Franca Ferraraccio, Michele D’Amico, Clara Di Filippo

**Affiliations:** 1 Department of Experimental Medicine, Division of Pharmacology, University of Campania, via S. Maria di Costantinopoli 16, 80138 Naples, Italy; dariosin@uab.edu (D.S.); mariaconsiglia.trotta2@unicampania.it (M.C.T.); brigida.annalisa@gmail.com (A.L.B.); rosa.maisto@unicampania.it (R.M.); clara.difilippo@unicampania.it (C.D.F.); 2 “Maria Guarino” Foundation—AMOR No Profit Association, 80078 Pozzuoli, Italy; margluon@gmail.com; 3 Department of Physical and Mental Health and Preventive Medicine, University of Campania, 80138 Naples, Italy; franca.ferraraccio@unicampania.it

**Keywords:** streptozotocin, ozone therapy, pancreas, rat

## Abstract

**Background:** The rat model of streptozotocin (STZ)-induced pancreatic damage was used to examine whether a systemic oxygen/ozone mixture could be beneficial for the pancreas by reducing the machinery of the local detrimental mediators released by STZ. **Results:** The results showed that oxygen/ozone administration (150 µg/Kg i.p.) for ten days in STZ rats increased the endogenous glutathione-s-transferase (GST) enzyme and nuclear factor-erythroid 2-related factor 2 (Nrf2) into the pancreatic tissue, together with reduction of 4-hydroxynonenal (4-HNE) and PARP-1 compared to STZ rats receiving O_2_ only. Interestingly, these changes resulted in higher levels of serum insulin and leptin, and pancreatic glucagon immunostaining. Consequently, glucose metabolism improved as evidenced by the monitoring of glycemia throughout. **Conclusions:** This study provides evidence that systemic administration of oxygen/ozone reduces the machinery of detrimental mediators released by STZ into the pancreas with less local damage and better functionality.

## 1. Introduction

Streptozotocin (STZ) is a glucose analogue molecule up-taken into the beta cells via the GLUT2 glucose transporter, and then split into glucose and N-methyl-N-nitrosourea (MNU) [1]. Due to the MNU alkylating properties, STZ causes a state of insulin-dependent diabetes due to biological macromolecule modifications, reactive oxygen species (ROS) generation, DNA fragmentation and beta cell disruption [1], most frequently caused by the accumulation of STZ-induced 4-hydroxynonenal (4-HNE) adducts within the pancreas, and therefore STZ is used as a diabetic inducer [2,3].

Recently, it has become widely accepted that one of the strategies to reduce the oxidative damage, blood stress and pancreas damage caused by STZ in rats is the administration of an oxygen/ozone mixture [4], even though its molecular mechanism has not been completely elucidated [5]. The therapeutic efficacy of oxygen/ozone may be induced by the controlled and moderate oxidative stress produced by the reaction of ozone with several biological components. The involvement of the nuclear factor-erythroid 2-related factor 2 (Nrf2), and the following release of numerous antioxidant enzymes, such as superoxide dismutase (SOD), glutathione peroxidase (GPx), glutathione-s-transferase (GST), catalase (CAT), heme-oxygenase-1 (HO-1), NADPH-quinoneoxidoreductase (NQO-1) [6,7] seems to play a master regulation of many cellular activities [8].

Assuming that oxygen/ozone treatment may be beneficial for the pancreas as earlier described by Martinez et al., (2005) [5], and that it is able to activate the general mechanism described by Sagai and Bocci (2011) [6] and Re et al., (2014) [7] in vivo, the aim of our study was to assess whether an oxygen/ozone mixture reduces the machinery of detrimental mediators released by STZ into the pancreatic tissue, such as the local formation of 4-HNE, PARP-1 cleavage and DNA strand break, when this diabetogenic chemical is used in vivo, ultimately leading tissue protection.

## 2. Materials and Methods

### 2.1. Animals

Healthy male Sprague Dawley rats (Harlan, San Pietro al Natisone, Udine, Italy) of four–six months and weighing about 180 ± 10 g, were housed in individual cages with controlled temperature (21–23 °C), lighting (12–12 h light-dark cycle), and humidity (55–60%). A standard chow pellet diet and water were administered *ad libitum*.

Ethics approval: All experimental procedures in accordance with Italian (Decree 116/92) and European Community (E.C. L358/1 18/12/86) Guidelines on the use and protection of laboratory animals. All efforts were taken to minimize animal suffering and reduce the number of animals used.

### 2.2. Ozone and STZ Treatments

Ozone (O_2_/O_3_ mixture) was generated with ozonator equipment from medical grade oxygen, the ozone concentration was measured using a UV spectrophotometer at 254 nm and it was administered immediately upon generation [9].

STZ was dissolved in 10 mM citrate buffer (pH 4.5), freshly prepared before injection and administered at 65 mg/Kg/10 mL i.p. [10,11].

### 2.3. Experimental Groups

A total of five groups were formed. One group consisted of five rats receiving citrate buffer only (CTR), without STZ administration, serving as the control for the group receiving STZ only. No differences between this control group with the following control group treated only by O_2_ (see below) were observed. A total of 15 rats received a single i.p. injection of 2 mL of STZ only. One week after the STZ injection, blood glucose levels were monitored (Glucometer Elite XL, Bayer Corp., Elkhart, IN, USA) with the aim of evaluating the insurgence of hyperglycemia. Blood glucose levels were monitored throughout the entire experimental protocol. Those rats developing hyperglycemia greater than 250 mg/dL were included in the study and divided into three groups. Of these, five rats were sacrificed in order to ascertain the pancreatic damage; five rats received a single injection of 1.5 mL of O_2_ i.p. once a day for seven days (STZ + O_2_); five rats received 1.5 mL of an oxygen/ozone (O_2_/O_3_) mixture (equivalent to 150 μg/kg) once a day for seven days i.p. (STZ + O_3_) [5,12,13]. A further group of five rats received only 1.5 mL of O_2_/O_3_ i.p. once a day for seven days and served as a control for the group receiving STZ + O_3_. A glycemia greater than 250 mg/dL was considered index of putative pancreas damage and continuously monitored through the study. At the end of the treatments (seven days after STZ injection), the animals were euthanized by an intramuscular injection of ketamine hydrochloride (100 mg/kg) and medetomidine (0.25 mg/kg). Afterwards, pancreas were promptly removed for biochemical and histological studies.

### 2.4. Tissue Sampling

Samples of the pancreatic tissue were taken. These were immediately cut in two halves; one was frozen at −80 °C for biochemical analyses and the other was paraffin-embedded and cut in 5 μm serial sections for histochemical staining with hematoxylin eosin or immunohistochemistry. 

### 2.5. Western Blotting Assay

Western blots were performed to evaluate the levels in pancreatic samples of Nuclear Factor Erythroid 2-Related Factor (Nrf2), and glutathione-s-transferase (GST) and Poly(ADP-ribose) polymerase-1 (PARP-1). Pancreatic tissues were minced into small pieces with a blender, then suspended in protein lysis buffer [HEPES 25 mM; EDTA 5 mM; SDS 1%; Triton X-100 1%; PMSF 1 mM; MgCl_2_ 5 mM; Protease Inhibitor Cocktail (Roche, Mannheim, Germany); Phospahatase Inhibitor Cocktail (Roche, Mannheim, Germany)] and centrifuged for 30 min at 4000× *g* at 4 °C [14]. Total proteins were quantized by the Bio-Rad protein assay (Bio-Rad Laboratories, Milan, Italy). Each protein sample was loaded and electrophoresed in a 8% polyacrylamide gel, and electrotransferred onto a PVDF membrane. Blots were blocked with 5% nonfat dry milk with PBS-T (PBS-0.05% Tween 20) for 1 h at room temperature and then incubated overnight at 4 °C with primary specific antibodies in blocking solution (anti-Nrf2 (T-19) (1:200; sc-30915 Santa Cruz Biotech, Santa Cruz, CA, USA), anti-GST (1:1000, ab19256 Abcam, Cambridge, UK), anti-PARP-1 (A-20) (1:200; sc-1562 Santa Cruz Biotech, CA, USA)). Immunoreactive signals were detected with goat anti-rabbit (sc-2004) and anti-goat IgG (sc-2020) horseradish peroxidase-conjugated secondary antibody (1:2000 in PBS-T; Santa Cruz Biotech, CA, USA, incubation time: 1 h at room temperature). The signal was visualized using an ECL system (Amersham Pharmacia, Uppsala, Sweden). The semi-quantitative analysis of protein levels was performed by the ChemiDoc-It 5000, using VisionWorks Life Science Image Acquisition and Analysis software (UVP, Upland, CA, USA). The signals were expressed as densitometric units (DU). Protein levels were normalized with respect to the signal obtained with anti-actin (C-2) (1:200 in blocking solution; sc-8432, Santa Cruz Biotech, CA, USA, overnight at 4 °C).

### 2.6. Immunohistochemistry

After the pancreatic samples were paraffin-embedded and cut in 5 μm serial sections, the paraffin was removed by a xylene substitute (Hemo-De; Thermo-Fisher Scientific, Darmstadt, Germany). The immunohistochemistry procedure was performed by BenchMark Automated IHC/ISH slide staining system, according to manufacturer’s instructions (BenchMark Ventana, Tucson, AZ, USA). Briefly, tissue sections were sequentially rehydrated with ethanol gradient washes, pre-heated, and stained with hematoxylin and eosin. Citrate antigen retrieval was performed by placing slides in citrate buffer (0.1 M citric acid monohydrate, and 0.1 M sodium citrate; pH 6) in a water filled steamer for 20 min. Endogenous peroxidase activity was quenched in 3% hydrogen peroxide aqueous solution for 15 min and non-specific antibody binding was inhibited by incubation for 1 h at room temperature in blocking solution (1% BSA, 0.2% powdered skim milk, 0.3% Triton-X 100 in PBS). Sections were incubated with specific anti-insulin (1:100 in blocking solution; ab-7842, Abcam, UK) and anti-glucagon (1:100 in blocking solution; ab-10988, Abcam, UK) antibodies, washed with PBS, incubated with biotin-conjugated secondary antibodies and avidin-biotin peroxidase complex (DBA, Milan, Italy), and 3,3′diaminoenzidine (DAB) reaction was used to locate the specific antigens in each section. Slides were counterstained with haematoxylin. Immunostaining was analyzed by an expert pathologist (intraobserver variability 5%). Measuring and calculating antigenic expression were done by Leica IM500 and statistics program Leica QWIN (Leica, Wetzlar, Germany). Four distinct preparations for each pancreatic sample were done and 20 fields of view were analyzed in each preparation for a total area of 1.43012e ± 0.001 μm^2^ for ×400 magnification.

### 2.7. Immunofluorescence

Pancreatic tissue sections were rehydrated, washed with PBS and incubated 1 h with blocking solution 5% bovine serum albumin (BSA) (Sigma-Aldrich, St. Louis, MO, USA) 0.05% Tween in PBS (PBS-T), and then incubated overnight at 4 °C with monoclonal anti-RAD51 antibodies (1:100 in blocking solution; ab-88572, Abcam, UK). Alexa Fluor ^®^ 488 (Jackson Laboratory, West Baltimore Pike, West Grove, PA, USA)-conjugated goat polyclonal antibody (1:1000 in PBS-T; 1 h at room temperature) was used as secondary for Rad51 detection. Nuclei were counterstained by DAPI. Quantification of fluorescence intensity was determined by Leica software (Leica, Wetzlar, Germany).

### 2.8. 4-Hydroxynonenal, Insulin and Leptin Levels

Using Enzyme Linked Immunosorbent Assay (ELISA) test, pancreatic levels of 4-Hydroxynonenal (MBS736336 MyBiosource—Merk Millipore, Milan, Italy) and serum levels of insulin (EZRMI-13K Merk Millipore, Italy) and leptin (EZRL-83K Merk Millipore, Italy) were quantified according to the manufacturer’s instructions.

### 2.9. Statistical Analysis

Data values are expressed as mean ± s.e.m. One-way ANOVA followed by Dunnett’s test was used in order to assess the variance among the groups. The alpha critical value was set as less than 0.05 to be considered significant.

## 3. Results

### 3.1. STZ Induces Pancreatic Tissue Damage

**Figure 1:**
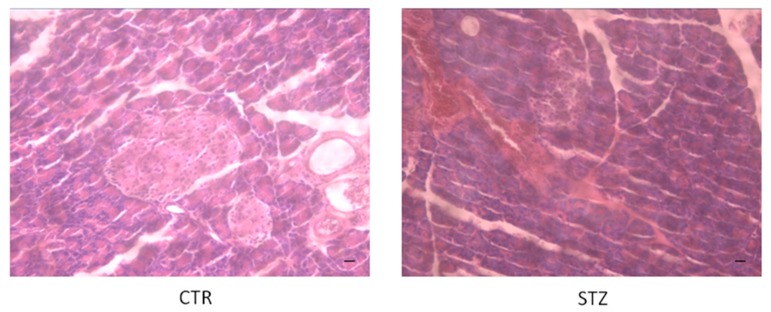
Hematoxylin and eosin (HE) staining. HE of pancreatic tissue from untreated and STZ rats at seven days. CTR = saline-treated rats; STZ = STZ (65 mg/kg/10 mL)-treated rats. Scale bar = 50 µm.

Histochemical analysis with hematoxylin and eosin showed pancreatic tissue damage after seven days post-STZ administration (Figure 1). In particular, CTR rats showed normal acini and normal cellular population in islets of Langerhans and absence of damage, while STZ-treated rats showed a induced lesion of pancreatic tissue with reduced islet numbers and size after seven days (Figure 1). 

#### Ozone Reduces 4-HNE Adduct in STZ Rats

**Figure 2:**
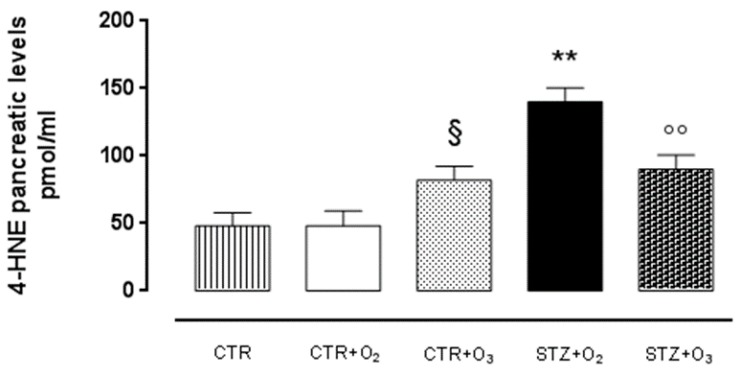
4-HNE pancreatic levels. ELISA test results of 4-HNE pancreatic levels (pmol/mL ± s.e.m.), after one week of STZ: ozone-treated STZ rats show a significant reduction in pancreatic 4-HNE levels compared to O_2_-treated STZ rats (*p* < 0.01). CTR = saline-treated rats; CTR + O_2_ = oxygen-treated rats; CTR + O_3_ = ozone-treated rats; STZ + O_2_ = oxygen-treated STZ rats; STZ + O_3_ = ozone-treated STZ rats. Results are the mean ± s.e.m. of five experiments. § *p* < 0.05 vs. CTR + O_2_; ** *p* < 0.01 vs. CTR + O_3_; °° *p* < 0.01 vs. STZ + O_2_.

After one week of STZ, 4-HNE levels were significantly increased (STZ + O_2_) with respect to O_2_ only (CTR + O_2_) (Figure 2). This increase was maximal seven days after STZ. O_2_/O_3_ injection (STZ + O_3_) showed significant reduction of 4-HNE levels compared to STZ rats administered only with O_2_ (STZ + O_2_) (*p* < 0.01) (Figure 2). 

### 3.2. Ozone Treatment Reduces Pancreatic Tissue Damage in STZ Rats

**Figure 3:**
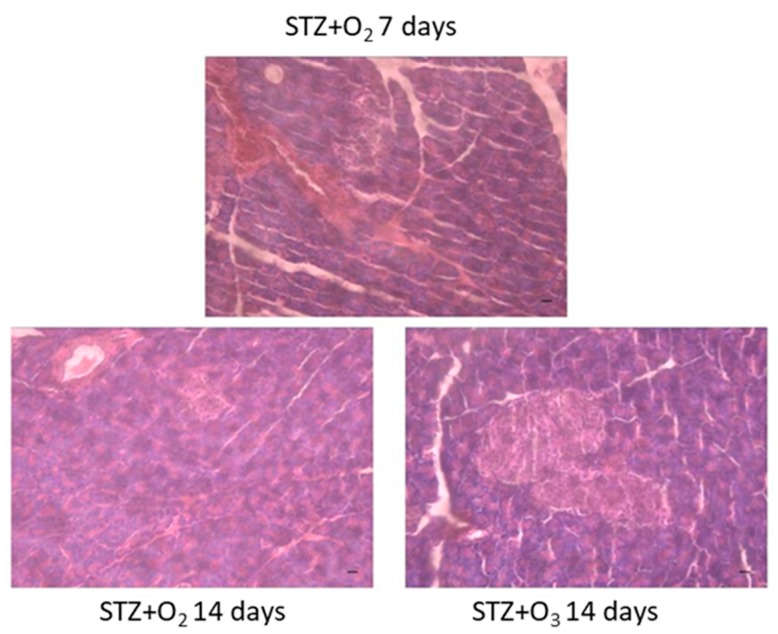
Hematoxylin and eosin (HE) staining. HE of pancreatic tissue from STZ rats treated with O_2_ seven days post STZ and 14 days post STZ. Pancreatic tissue damage was present at seven days and maximal at 14 days. Ozone treatment showed restoration of normal cellular population size of islets of Langerhans and absence of islet damage. STZ + O_2_ = oxygen-treated STZ rats; STZ + O_3_ = oxygen/ozone-treated STZ rats. Scale bar = 50 µm.

If observed after a further seven days of hyperglycemia, the rats receiving STZ alone showed a further degeneration of the pancreatic tissue compared to the damage shown seven days post-STZ injection. This was characterized by decrease of the number of islets and worse damage (Figure 3). Ozone treatment rescued the pancreatic tissue by the STZ-induced damage at this time point, reducing the tissue degeneration evidenced by the partial restoration of normal cellular population size of islets of Langerhans and absence of islet damage (Figure 3).

#### 3.2.1. Ozone Treatment Reduces Beta Cells Death and DNA Damage in STZ Rats

**Figure 4:**
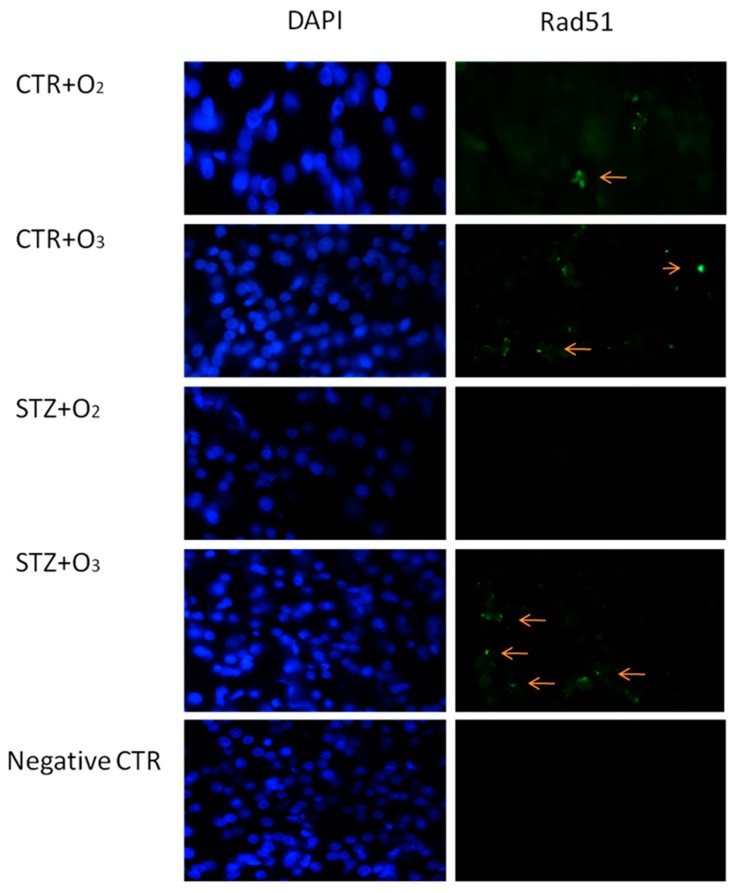
RAD51 immuno-positive signals. DAPI column shows DAPI nuclear staining (blue); Rad51 column shows the same DAPI-stained cells expressing Rad51 protein (green). Ozone treatment was able to increase Rad51 immuno-profiles (red arrows). CTR + O_2_ = oxygen-treated rats; CTR + O_3_ = ozone-treated rats; STZ + O_2_ = oxygen-treated STZ rats; STZ + O_3_ = ozone-treated STZ rats. Negative CTR: Negative control (without primary antibody) for staining in order to confirm antibody specificity.

**Figure 5:**
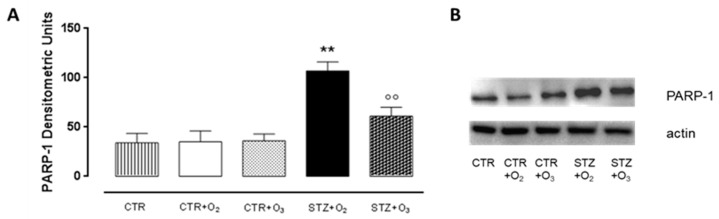
PARP-1 pancreatic levels. Western blot analysis of PARP-1 pancreatic levels after one week of STZ: PARP-1 levels were significantly reduced in STZ rats administered with 10 treatments of 150 μg/kg of O_2_/O_3_ mixture compared to STZ rats administered with 1.5 mL O_2_ (*p* < 0.01). Results are expressed as densitometric units ± s.e.m. of five experiments. CTR = saline-treated rats; CTR + O_2_ = oxygen-treated rats; CTR + O_3_ = ozone-treated rats; STZ + O_2_ = oxygen-treated STZ rats; STZ + O_3_ = ozone-treated STZ rats. ** *p* < 0.01 vs. CTR + O_3_; °° *p* < 0.01 vs. STZ + O_2_.

Immunofluorescence analysis showed that ozone treatment significantly reduced cell death, shown by DAPI labeling, as observed after ten days of treatment (Figure 4). Ozone treatment also decreased DNA damage, as evidenced by increased Rad51 into the tissue (Figure 4), paralleled by reduced PARP-1 expression (Figure 5).

#### 3.2.2. Ozone Treatment Increases Nrf2 and GST Pancreatic Levels in STZ Rats

**Figure 6:**
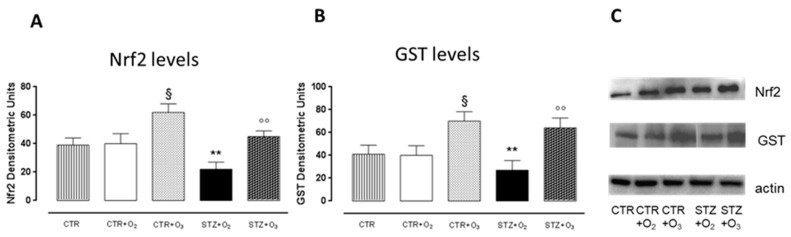
Nrf2 and GST pancreatic levels. Western blot analysis after one week of STZ: ozone treatment significantly increased Nrf2 and GST expression levels in STZ rats (*p* < 0.01) compared to untreated STZ rats. C: representative protein bands of western blot for Nrf2, GST and actin. Results are expressed as densitometric units ± s.e.m. of five experiments. CTR = saline-treated rats; CTR + O_2_ = oxygen-treated rats; CTR + O_3_ = ozone-treated rats; STZ + O_2_ = oxygen-treated STZ rats; STZ + O_3_ = ozone-treated STZ rats. § *p* < 0.05 vs. CTR + O_2_; ** *p* < 0.01 vs. CTR + O_3_; °° *p* < 0.01 vs. STZ + O_2_.

STZrats administered only with oxygen had significantly (*p* < 0.01) low levels of Nrf2 and GST as evidenced by Western blot with respect to oxygen alone (CTR + O_2_). These markers were significantly increased in ozone-treated STZ rats (STZ + O_3_) towards the values expressed in the O_2_ group (STZ + O_2_) (Figure 6).

#### 3.2.3. Ozone Reduces Glucagon and Increases Insulin Levels in Pancreas of STZ Rats

**Figure 7:**
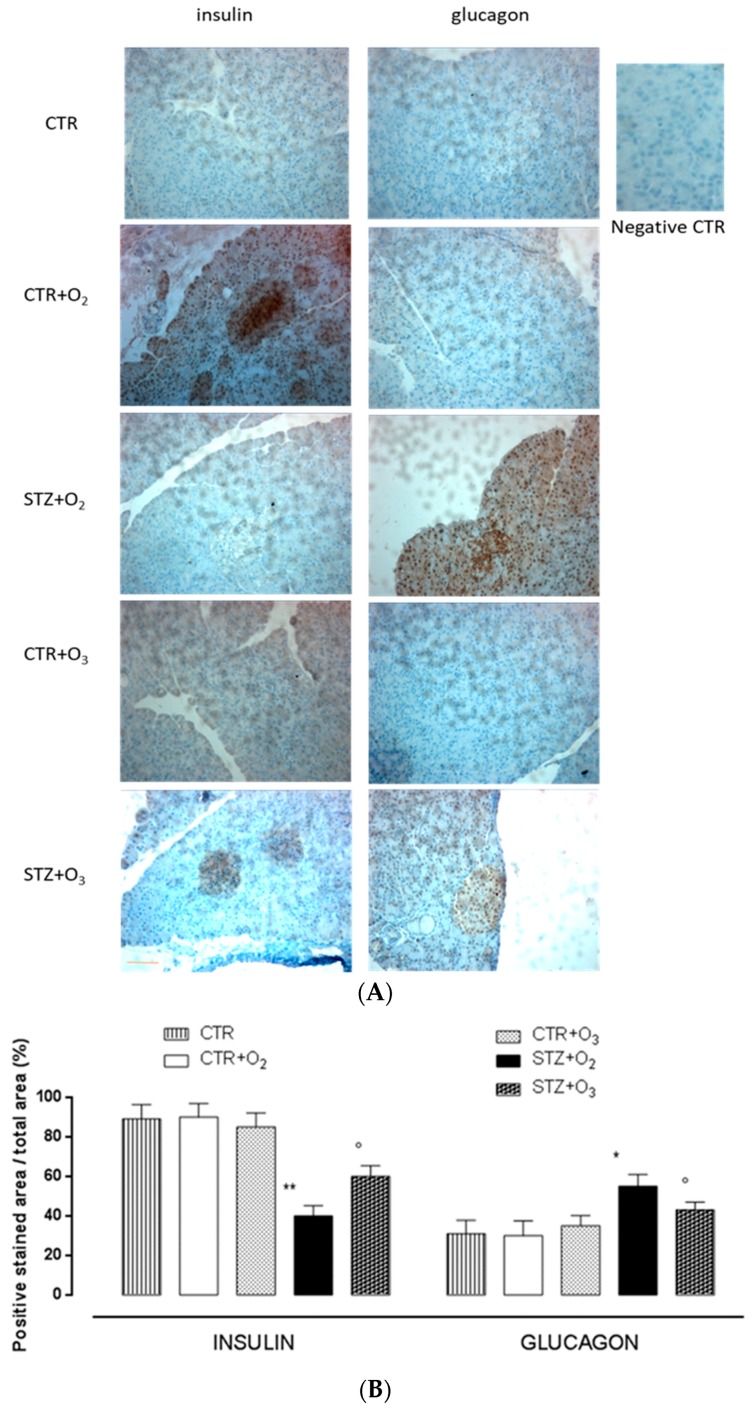
Insulin and glucagon immunostaining. (**A**) The picture shows insulin and glucagon immunostaining in pancreatic tissues. Scale-bar: 50 µm. (**B**) Results are expressed as positive stained area ± s.e.m. CTR = saline-treated rats; CTR + O_2_ = oxygen-treated rats; CTR + O_3_ = ozone-treated rats; STZ + O_2_ = oxygen-treated STZ rats; STZ + O_3_ = ozone-treated STZ rats. CTR + O_2_ = oxygen-treated rats; CTR + O_3_ = ozone-treated rats; STZ + O_2_ = oxygen-treated STZ rats; STZ + O_3_ = ozone-treated STZ rats. * *p* < 0.05 vs. CTR + O_2_; ** *p* < 0.01 vs. CTR + O_2_; ° *p* < 0.01 vs. STZ + O_2_. The insert represents the negative CTR: Negative control (without primary antibody) for staining in order to confirm antibody specificity.

Glucagon levels were significantly increased in oxygen-treated STZ rats (STZ + O_2_) (*p* < 0.01) compared to oxygen rats (CTR + O_2_) (Figure 7). In STZ rats, O_2_/O_3_ treatment (150 μg/kg i.p.) significantly decreased glucagon levels (*p* < 0.01) compared to STZ + O_2_ and resulted in values toward those of CTR + O_2_ (Figure 7). Immunohistochemical analysis showed also that ozone treatment increased insulin by itself and if associated with STZ, this increase was lower than ozone alone (Figure 7).

#### 3.2.4. Ozone Treatment Improves Insulin and Leptin Serum Levels, Reducing Glycemia in STZ Rats

**Figure 8:**
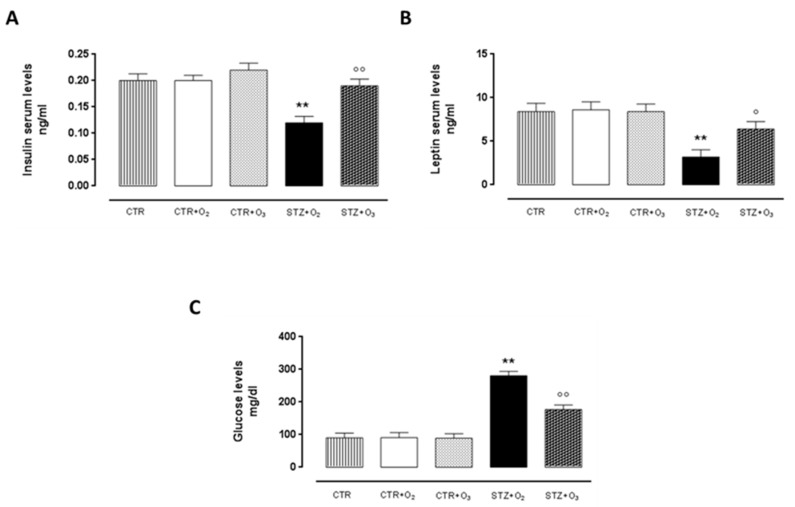
Insulin, leptin and glycemia levels. ELISA test results of insulin and leptin serum levels (mg/dl ± s.e.m.), one week after STZ. Ozone treatment significantly increased insulin and leptin levels, that were low in STZ rats. Ozone treatment significantly reduced the high glycemia levels in STZ rats (*p* < 0.01). CTR = saline-treated rats; CTR + O_2_ = oxygen-treated rats; CTR + O_3_ = ozone-treated rats; STZ + O_2_ = oxygen-treated STZ rats; STZ + O_3_ = ozone-treated STZ rats. ** *p* < 0.01 vs. CTR + O_2_; ° *p* < 0.05 vs. STZ + O_2_; °° *p* < 0.01 vs. STZ + O_2_.

In STZ + O_2_ rats both insulin and leptin serum levels were significantly decreased (*p* < 0.05) compared to control rats with O_2_ only (Figure 8). Ozone treatment partially restored insulin (*p* < 0.05) and leptin levels (*p* < 0.01) in STZ rats. Interestingly, glycemia was significantly reduced in ozone-treated STZ rats (STZ + O_3_) (*p* < 0.01) with respect to oxygen-treated STZ rats (STZ + O_2_) (Figure 8).

## 4. Discussion

The results presented here suggest that oxygen/ozone is a preservative tool for pancreatic cell death caused by the diabetogenic chemical streptozotocin. The proposed mechanism involves a decreased release of the STZ-induced alkenal 4-HNE within the pancreas with less local damage, DNA strand breakage, as well as increased activity of the Nrf2-GST pathway and increased final pancreas functionality [15]. 

The efficacy of ozone in several pathologic conditions has already been extensively described [16]. Its use includes adjuvant therapeutic treatment of severe chronic pathologies, such as cardiovascular diseases, chronic obstructive pulmonary diseases, neuropathic pain, multiple sclerosis, the dry form of age-related macular degeneration and prevention of uterine adhesions [9,16,17,18]. Our previous works have substantially contributed to the oxygen/ozone literature since the pioneering studies of Di Filippo et al. in 2008 [12], that showed the efficacy of ozone treatment in oxidation- and inflammation-related pathologies. Ozone has been proved to have beneficial effects in rats during myocardial reperfusion [10] and, more recently, in rats subjected to prolonged high-intensity physical exercise, reducing muscular fatigue and improving cardiac performance [11]. 

The molecular mechanisms underlying these ozone-related phenomena encompass several mediators and molecules involved in the oxidative and inflammatory responses [19]. The ozone research, however, further deserves insights. Nowadays, it is well-accepted that the hormetic action of oxygen/ozone on cells, a process that ameliorates and improves cellular stress resistance, survival and longevity in response to low stress levels [20]. Overall, low doses of oxygen/ozone induce a small oxidative stress signal that activates antioxidant responses [21], quenching the oxidative damage and promoting the release of resolution mediators. Among these, the nuclear factor-erythroid 2-related factor 2 (Nrf2) is a leucine zipper transcription factor that, when properly activated, is able to restore the cell redox homeostasis by increasing the production of several antioxidant enzymes, such as glutathione-s-transferase (GST) [6,7,8,22]. We show here that the repeated administration of low oxygen/ozone doses to rats induces the pancreas to release high levels of Nrf2 within tissue, accompanied by high levels of GST. GST, highly expressed after the transcription of antioxidant response elements (ARE) by Nrf2 [6], is one of the main enzymes degrading an α,β-unsaturated hydroxyalkenal adduct, the 4-hydroxynonenal (4-HNE) [2], highly produced in STZ rats and possibly highly damaging for cells and tissues [3,23]. Interestingly, the level of this adduct has been found to be deeply low after oxygen/ozone treatment of STZ rats compared to STZ rats treated with O_2_ only. Considering that pancreatic beta cells physiologically show low levels of antioxidant enzyme gene expression and are deeply influenced by ROS [1], an interesting hypothesis is that ozone, as a low-stressing molecule, once administered to rats, promotes a low stress preconditioning status of the pancreatic tissue, a phenomenon called hormesis [22,24], which in turn is able to increase and activate the Nrf2-GST pathway, quenching the STZ-induced release of 4-HNE into the tissue, with the end point being cell survival. 

It has been demonstrated that ozone therapy decreases the expression of apoptotic genes and DNA fragmentation in the nephropathic kidney tissue of diabetic rats [25]. Another interesting hypothesis and novelty of the present study is that ozone influences the expression and levels of the nuclear enzyme poly(ADP-ribose) polymerase-1 (PARP-1) within the pancreatic cells. It is noteworthy that STZ induces DNA strand breaks and poly(ADP-ribose) polymerase in pancreatic islets, depleting intracellular NAD levels and inhibiting proinsulin synthesis [26,27]. PARP-1 is an enzyme that attempts to repair DNA breakage following a noxius stimulus [28], and its absence prevents beta cell death mediated by STZ in knockout mice [29,30,31]. Indeed, exacerbated PARP-1 activation results in glycolytic inhibition through ATP depletion and glyceraldehyde-3-phosphate dehydrogenase (GAPDH) blocking [32], influencing in this way the glucose metabolism. PARP-1 inhibition shows ameliorative effects in STZ-diabetic nephropathy [33], and, in line with this, the injection of nicotinamide or other PARP inhibitors, in parallel or prior to STZ administration, protects the beta cells against the toxic action of STZ, preventing the development of a diabetic state [34,35]. Noteworthy, ROS-mediated PARP-1 over-activation is able to induce cell death by necrosis [36]; furthermore, it has been demonstrated that in a mouse model of type 1 diabetes, beta cells die by necrosis in a process of programmed cell death that requires both apoptosis and necroptosis [37]. Following oxygen/ozone treatment, the pancreas of STZrats showed lower levels of PARP-1 with respect to STZrats without oxygen/ozone, suggesting less DNA breakage. These reduced levels of PARP-1 were accompanied by higher levels of the DNA repair protein RAD51. This protein is well recognized as a DNA repair protein involved in the homologous recombination of DNA [38], thus promoting cell survival. The molecular machinery activated by oxygen/ozone was translated functionally into improvement of serum insulin and leptin levels in STZrats, resulting in an overall reduction of glycemia.

## 5. Conclusions

**Figure 9:**
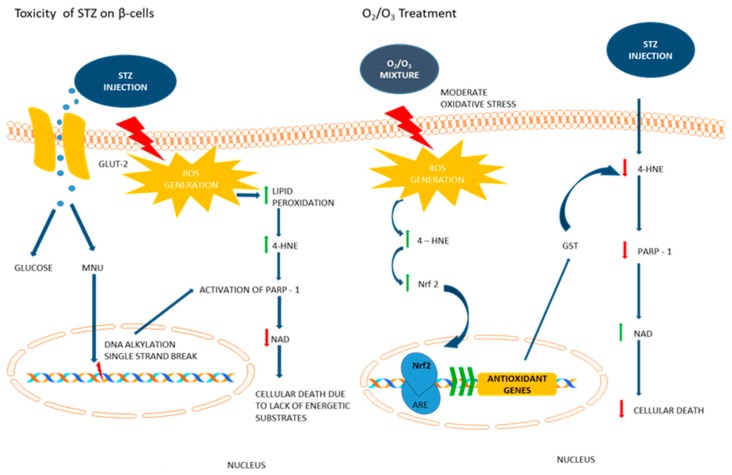
Proposed mechanism of ozone activity in STZ rats. Left panel: STZ induces increased reactive oxygen species (ROS) production, lipid peroxidation, accumulation of 4-hydroxynonenal (4-HNE) and DNA fragmentation; consequently, PARP-1 levels are increased in order to attempt DNA repair. Right panel: oxygen/ozone administration promotes a moderate oxidative preconditioning state, which in turn is able to increase Nrf2, GST production and anti-oxidative defense mechanisms. 4-HNE, PARP-1 and cellular death are decreased, this reduction resulted in higher levels of insulin and leptin and consequently improved the glycemia.

In conclusion, ozone treatment improves pancreatic cell survival. This was exerted through the increase of the endogenous Nrf2, GST enzymes and the consequent reduction of 4-HNE and PARP-1 levels in pancreatic tissues (Figure 9). This reduction results in higher levels of insulin and leptin and the consequent improvement of glucose metabolism. From the translational point of view, this latter improvement may be beneficial in preventing long-term diabetes-induced organ damage.

## References

[1] Lenzen S., Drinkgern J., Tiedge M. (1996). Low antioxidant enzyme gene expression in pancreatic islets compared with various other mouse tissues. Free Radic. Biol. Med..

[2] Traverso N., Menini S., Odetti P., Pronzato M.A., Cottalasso D., Marinari U.M. (2002). Diabetes impairs the enzymatic disposal of 4-hydroxynonenal in rat liver. Free Radic. Biol. Med..

[3] Lupachyk S., Shevalye H., Maksimchyk Y., Drel V.R., Obrosova I.G. (2011). PARP inhibition alleviates diabetes-induced systemic oxidative stress and neural tissue 4-hydroxynonenal adduct accumulation: Correlation with peripheral nerve function. Free Radic. Biol. Med..

[4] Al-Dalain S.M., Martinez G., Candelano-Jali E., Menéndez S., Re L., Giuliani A., León O.S. (2001). Ozone treatment reduces markers of oxidative and endothelial damage in an experimental diabetes model in rats. Pharmacol. Res..

[5] Martìnez G., Al-Dalain S.M., Menendez S., Giuliani A., Léon O.S. (2005). Ozone treatment reduces blood oxidative stress and pancreas damage in streptozotocin induced diabetes model in rats. Acta Farm. Bonaer..

[6] Sagai M., Bocci V. (2011). Mechanisms of Action Involved in Ozone Therapy: Is healing induced via a mild oxidative stress?. Med. Gas Res..

[7] Re L., Martínez-Sánchez G., Bordicchia M., Malcangi G., Pocognoli A., Morales-Segura M.A., Rothchild J., Rojas A. (2014). Is ozone pre-conditioning effect linked to Nrf2/EpRE activation pathway in vivo? A preliminary result. Eur. J. Pharmacol..

[8] Hybertson B.M., Gao B., Bose S.K., McCord J.M. (2011). Oxidative stress in health and disease: The therapeutic potential of Nrf2 activation. Mol. Asp. Med..

[9] Fuccio C., Luongo C., Capodanno P., Giordano C., Scafuro M.A., Siniscalco D., Lettieri B., Rossi F., Maione S., Berrino L. (2009). A single subcutaneous injection of ozone prevents allodynia and decreases the over-expression of pro-inflammatory caspases in the orbito-frontal cortex of neuropathic mice. Eur. J. Pharmacol..

[10] Li H., Liu Z., Wang J., Wong G.T., Cheung C.W., Zhang L., Chen C., Xia Z., Irwin M.G. (2013). Susceptibility to myocardial ischemia reperfusion injury at early stage of type 1 diabetes in rats. Cardiovasc. Diabetol..

[11] Rossi S., Maisto R., Gesualdo C., Trotta M.C., Ferraraccio F., Kaneva M.K., Getting S.J., Surace E., Testa F., Simonelli F. (2016). Activation of Melanocortin Receptors MC1 and MC5 Attenuates Retinal Damage in Experimental Diabetic Retinopathy. Mediat. Inflamm..

[12] Di Filippo C., Marfella R., Capodanno P., Ferraraccio F., Coppola L., Luongo M., Mascolo L., Luongo C., Capuano A., Rossi F. (2008). Acute oxygen ozone administration to rats protects the heart from ischemia reperfusion infarct. Inflamm. Res..

[13] Di Filippo C., Trotta M.C., Maisto R., Siniscalco D., Luongo M., Mascolo L., Alfano R., Accardo M., Rossi C., Ferraraccio F. (2015). Daily Oxygen/O_3_ Treatment Reduces Muscular Fatigue and Improves Cardiac Performance in Rats Subjected to Prolonged High Intensity Physical Exercise. Oxid. Med. Cell. Longev..

[14] Siniscalco D., Sapone A., Giordano C., Cirillo A., de Magistris L., Rossi F., Fasano A., Bradstreet J.J., Maione S., Antonucci N. (2013). Cannabinoid receptor type 2, but not type 1, is up-regulated in peripheral blood mononuclear cells of children affected by autistic disorders. J. Autism Dev. Disord..

[15] Lenzen S. (2008). The mechanisms of alloxan- and streptozotocin-induced Diabetes. Diabetologia.

[16] Zanardi I., Borrelli E., Valacchi G., Travagli V., Bocci V. (2016). Ozone: A Multifaceted Molecule with Unexpected Therapeutic Activity. Curr. Med. Chem..

[17] Uysal B., Demirbag S., Poyrazoglu Y., Cayci T., Yesildaglar N., Guven A., Sürer I., Korkmaz A. (2012). Medical ozone therapy decreases postoperative uterine adhesion formation in rats. Arch. Gynecol. Obstet..

[18] Borrelli E., Bocci V. (2013). Visual improvement following ozonetherapy in dry age related macular degeneration; a review. Med. Hypothesis Discov. Innov. Ophthalmol..

[19] Bocci V., Di Paolo N. (2009). Oxygen-ozone therapy in medicine: An update. Blood Purif..

[20] Bocci V.A., Zanardi I., Travagli V. (2011). Ozone acting on human blood yields a hormetic dose-response relationship. J. Transl. Med..

[21] Bocci V. (2012). How a calculated oxidative stress can yield multiple therapeutic effects. Free Radic. Res..

[22] Bocci V., Valacchi G. (2015). Nrf2 activation as target to implement therapeutic treatments. Front. Chem..

[23] Ji C., Amarnath V., Pietenpol J.A., Marnett L.J. (2001). 4-hydroxynonenal induces apoptosis via caspase-3 activation and cytochrome c release. Chem. Res. Toxicol..

[24] Calabrese E.J., Baldwin L.A. (2003). The hormetic dose-response model is more common than the threshold model in toxicology. Toxicol. Sci..

[25] Güçlü A., Erken H.A., Erken G., Dodurga Y., Yay A., Özçoban Ö., Şimşek H., Akçılar A., Koçak F.E. (2016). The effects of ozone therapy on caspase pathways, TNF-α, and HIF-1α in diabetic nephropathy. Int. Urol. Nephrol..

[26] Yamamoto H., Uchigata Y., Okamoto H. (1981). Streptozotocin and alloxan induce DNA strand breaks and poly(ADP-ribose) synthetase in pancreatic islets. Nature.

[27] Uchigata Y., Yamamoto H., Kawamura A., Okamoto H. (1982). Protection by superoxide dismutase, catalase, and poly(ADPribose) synthetase inhibitors against alloxan- and streptozotocin induced islet DNA strand breaks and against the inhibition of proinsulin synthesis. J. Biol. Chem..

[28] Islam B.U., Habib S., Ahmad P., Allarakha S., Ali A.M. (2015). Pathophysiological Role of Peroxynitrite Induced DNA Damage in Human Diseases: A Special Focus on Poly(ADP-ribose) Polymerase (PARP). Indian J. Clin. Biochem..

[29] Burkart V., Wang Z.Q., Radons J., Heller B., Herceg Z., Stingl L., Wagner E.F., Kolb H. (1999). Mice lacking the poly(ADP-ribose) polymerase gene are resistant to pancreatic beta-cell destruction and diabetes development induced by streptozocin. Nat. Med..

[30] Masutani M., Suzuki H., Kamada N., Watanabe M., Ueda O., Nozaki T., Jishage K., Watanabe T., Sugimoto T., Nakagama H. (1999). Poly(ADP-ribose) polymerase gene disruption conferred mice resistant to streptozotocin induced diabetes. Proc. Natl. Acad. Sci. USA.

[31] Pieper A.A., Brat D.J., Krug D.K., Watkins C.C., Gupta A., Blackshaw S., Verma A., Wang Z.Q., Snyder S.H. (1999). Poly(ADP-ribose) polymerase-deficient mice are protected from streptozotocin induced diabete. Proc. Natl. Acad. Sci. USA.

[32] Devalaraja-Narashimha K., Padanilam B.J. (2009). PARP-1 inhibits glycolysis in ischemic kidneys. J. Am. Soc. Nephrol..

[33] Shevalye H., Stavniichuk R., Xu W., Zhang J., Lupachyk S., Maksimchyk Y., Drel V.R., Floyd E.Z., Slusher B., Obrosova I.G. (2010). Poly(ADP-ribose) polymerase (PARP) inhibition counteracts multiple manifestations of kidney disease in long-term streptozotocin-diabetic rat model. Biochem. Pharmacol..

[34] Schein P.S., Cooney D.A., Vernon M.L. (1967). The use of nicotinamide to modify the toxicity of streptozotocin diabetes without loss of antitumor activity. Cancer Res..

[35] Kuchmerovska T., Shymanskyy I., Bondarenko L., Klimenko A. (2008). Effects of nicotinamide supplementation on liver and serum contents of amino acids in diabetic rats. Eur. J. Med. Res..

[36] Douglas D.L., Baines C.P. (2014). PARP1-mediated necrosis is dependent on parallel JNK and Ca^2+^/calpain pathways. J. Cell Sci..

[37] Zhao Y., Scott N.A., Fynch S., Elkerbout L., Wong W.W., Mason K.D., Strasser A., Huang D.C., Kay T.W., Thomas H.E. (2015). Autoreactive T cells induce necrosis and not BCL-2-regulated or death receptor-mediated apoptosis or RIPK3-dependent necroptosis of transplanted islets in a mouse model of type 1 diabetes. Diabetologia.

[38] Schultz N., Lopez E., Saleh-Gohari N., Helleday T. (2003). Poly(ADP-ribose) polymerase (PARP-1) has a controlling role in homologous recombination. Nucleic Acids Res..

